# The Indoor Fungus Cladosporium halotolerans Survives Humidity Dynamics Markedly Better than Aspergillus niger and Penicillium rubens despite Less Growth at Lowered Steady-State Water Activity

**DOI:** 10.1128/AEM.00510-16

**Published:** 2016-08-15

**Authors:** Frank J. J. Segers, Karel A. van Laarhoven, Hendrik P. Huinink, Olaf C. G. Adan, Han A. B. Wösten, Jan Dijksterhuis

**Affiliations:** a CBS-KNAW Fungal Biodiversity Centre, Applied and Industrial Mycology, Utrecht, The Netherlands; b Eindhoven University of Technology, Department of Applied Physics, Eindhoven, The Netherlands; c Utrecht University, Microbiology, Department of Biology, Utrecht, The Netherlands; HKI and University of Jena

## Abstract

Indoor fungi cause damage in houses and are a potential threat to human health. Indoor fungal growth requires water, for which the terms water activity (a_w_) and relative humidity (RH) are used. The ability of the fungi Aspergillus niger, Cladosporium halotolerans, and Penicillium rubens at different developmental stages to survive changes in a_w_ dynamics was studied. Fungi grown on media with high a_w_ were transferred to a controlled environment with low RH and incubated for 1 week. Growth of all developmental stages was halted during incubation at RHs below 75%, while growth continued at 84% RH. Swollen conidia, germlings, and microcolonies of A. niger and P. rubens could not reinitiate growth when retransferred from an RH below 75% to a medium with high a_w_. All developmental stages of C. halotolerans showed growth after retransfer from 75% RH. Dormant conidia survived retransfer to medium with high a_w_ in all cases. In addition, retransfer from 84% RH to medium with high a_w_ resulted in burst hyphal tips for Aspergillus and Penicillium. Cell damage of hyphae of these fungi after incubation at 75% RH was already visible after 2 h, as observed by staining with the fluorescent dye TOTO-1. Thus, C. halotolerans is more resistant to a_w_ dynamics than A. niger and P. rubens, despite its limited growth compared to that of these fungi at a lowered steady-state a_w_. The survival strategy of this phylloplane fungus in response to the dynamics of a_w_ is discussed in relation to its morphology as studied by cryo-scanning electron microscopy (cryo-SEM).

**IMPORTANCE** Indoor fungi cause structural and cosmetic damage in houses and are a potential threat to human health. Growth depends on water, which is available only at certain periods of the day (e.g., during cooking or showering). Knowing why fungi can or cannot survive indoors is important for finding novel ways of prevention. Until now, the ability of fungi to grow on media with little available water at steady state (unchanging conditions) has been important for evaluating whether a fungus can grow indoors. In the present study, we found that the fungus Cladosporium halotolerans, a common indoor fungus, is more resistant to changes in available water than the fungi Aspergillus niger and Penicillium rubens, despite the fact that the latter fungi can grow on media with low water availability. We concluded that the ability of fungi to deal with changes in humidity is at least as important as the ability to grow on low-water media.

## INTRODUCTION

People in Europe spend only 1.6 h a day outdoors (1), emphasizing the need for a healthy indoor environment (2). Indoor fungal growth represents a global problem. It has been estimated that about 25% of social housing in the European Union shows fungal growth. This not only causes disfigurement of the building materials but also poses a health threat (3, 4). Asthmatic and allergic patients are particularly at risk due to activation of the immune system by airborne fungal structures that are released from surface-grown fungal colonies (5–9). Mycotoxins produced by indoor fungi may also contribute to the health risk, but it is still unknown to what extent these toxic compounds are produced and released into the indoor environment (10, 11).

Highly diverse fungal species are present in the outdoor air, in particular species of Aspergillus, Penicillium, and Cladosporium (12). Their abundance in temperate climates is influenced by the seasons. For instance, Cladosporium is particularly prevalent during fall (13, 14). The abundance of fungal species in the indoor environment is affected by their predominance in the outdoor environment and by the indoor conditions (13–17). Penicillium chrysogenum and Aspergillus versicolor are particularly abundant after water damage or direct moistening, while Cladosporium occurs predominantly in indoor environments without water-related incidents (12, 18–22).

A lot of studies have been done on fungal growth in relation to water activity (a_w_) (23–25) and relative humidity (RH) (26, 27). Both a_w_ and RH express water's chemical potential, and therefore the availability of water. In this study, we use both terms, with the following relationship in equilibrium: RH = a_w_ × 100%.

Earlier studies which were done in an attempt to predict indoor fungal growth focused on the effects of RH and fungal growth on building materials, such as gypsum (28–31), wood (32–35), and concrete (36). Most fungi show growth in the range of 90 to 100% RH (12), but a restricted subset of fungi, such as Cladosporium sphaerospermum, P. chrysogenum, and A. versicolor, also grow on building materials, such as plywood, pine sapwood, and gypsum board, at a static RH as low as 75 to 80% (33). However, dynamic humidity of around 80% RH resulted in less-than-expected growth in the case of some materials, such as gypsum board (33, 37). This value of 80% RH is stated as the lower limit for indoor fungal growth. Indoor RH is dynamically averaged to be around 50%, thus raising the question of how fungal cells respond to periods of lower RH (38). Survival of fully developed colonies of Penicillium rubens in response to a_w_ dynamics was reported by Bekker (29). The effect of dynamic RH on the fungi Penicillium brevicompactum, Alternaria tenuissima, and Trichoderma harzianum in wood samples was studied by Li and Wadsö (35). They measured more heat release due to increased metabolic activity of fungal cells following an increase of RH by using an isothermal calorimeter. The impact of dynamic water availability on fungal growth was also studied in a model system of moistened building material that was dried and moistened again (22). The fungal growth seen in that study was due to the moisture content in the building material. The relevance of moisture content was shown in a study done by van Laarhoven et al. (31). Gypsum tablets equilibrated at a certain RH or soaked in a glycerol solution with the corresponding a_w_ were inoculated with P. rubens. The soaked tablets that had a higher moisture content showed markedly faster elongation of hyphae. However, there is a scarcity of knowledge on the effects of humidity changes on the cellular level.

The purpose of this study was to compare the responses of indoor fungi, namely, Aspergillus niger, Cladosporium halotolerans, and P. rubens, at different developmental stages to steady-state and dynamic water activity. C. halotolerans had markedly better survival at both increasing and decreasing a_w_ than that of P. rubens and A. niger. This difference in survival at dynamic a_w_ was despite the growth limit of C. halotolerans at a higher steady-state a_w_ than that for P. rubens and A. niger.

## MATERIALS AND METHODS

### Organisms and growth conditions.

A. niger N402 (39), C. halotolerans CBS 139586 (40), and P. rubens CBS 401.92 (28) were used in this study. The P. rubens strain was first identified as Penicillium chrysogenum but was later reclassified (41). Strains were routinely grown at 25°C on dichloran-18% glycerol agar (DG18 agar; Oxoid) (a_w_ = 0.96) (42, 43).

### Growth at steady-state a_w_.

Measurements of the growth of fungi at lower steady-state a_w_ were done as described by Segers et al. (40). Fungi were grown on malt extract agar (MEA) complemented with 0 to 50% glycerol (a_w_ of 0.99 to 0.75) to assess the lower limits of growth with respect to a_w_. The a_w_ values for the glycerol-agar mixtures were determined before and after growth experiments by using a Novasina labmaster-a_w_ instrument (Novasina, Lachen, Switzerland) as also described by Segers et al. (40). Cultures were inoculated with 3 μl of a spore solution containing 1 × 10^6^ conidia ml^−1^. These conidia were harvested from a 7-day-old culture by use of a T spatula (VWR, Amsterdam, The Netherlands), using ice-cold sterile 10 mM *N*-(2-acetamido)-2-aminoethanesulfonic acid, 0.02% Tween 80 (ACES; pH 6.8). The colony diameter was measured 3 times a week for 3 weeks. The growth speed was determined from the regression coefficients based on graphs of each colony.

### Survival during periods of dynamic a_w_.

**FIG 1:**
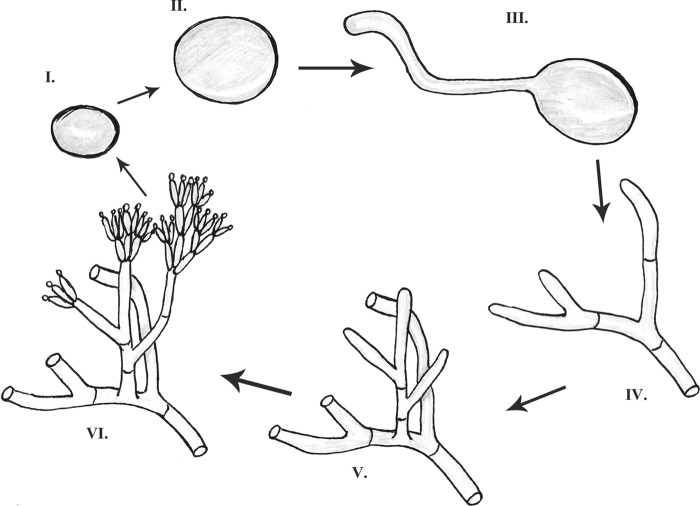
Schematic overview of the developmental stages of the indoor fungi used in this study, i.e., dormant conidia (I), swollen conidia (II), germlings (III), and mycelia without aerial hyphae (IV), with aerial hyphae (V), and with conidium-forming conidiophores (VI).

**FIG 2:**
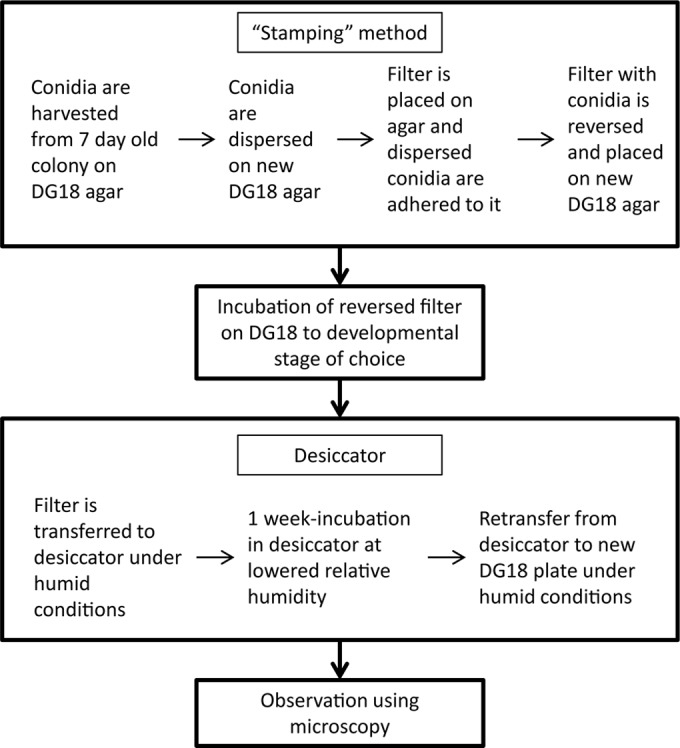
Flowchart of the experimental setup to study survival of indoor fungi after periods of lowered relative humidity.

To study survival of fungal cells during changes in a_w_, DG18 plates were inoculated with 50 μl of spore solution (1 × 10^6^ conidia ml^−1^). The inoculum was homogeneously spread on the agar surface by use of a T spatula and left to dry. A polycarbonate (PC) filter (47-mm diameter, 0.1-μm pore size; GE Water & Process Technologies) was placed on the surface of the inoculated agar to pick up the spores. Filters were immediately transferred to a new DG18 plate, with the side containing the spores oriented upwards. This “stamping” method resulted in 100 to 500 dispersed conidia per filter. The filters containing conidia were incubated in the dark on DG18 agar for 0 to 50 h, until the developmental stage of interest (Table 1; Fig. 1), as confirmed by light microscopy. Filters were removed from the agar plate and transferred to glass desiccators (6 liters) containing 150 to 300 ml saturated potassium chloride (RH, 84.3% ± 0.2%), sodium chloride (RH, 75.3% ± 0.1%), sodium bromide (RH, 57.6% ± 0.4%), or magnesium chloride (RH, 32.8% ± 0.2%) to control relative humidity (44). The filters were transferred to and from the desiccators within a humid environment (RH of >85%) to prevent exposure to low RH during transfer. The RH was quantified using a Testo 174H hygrometer (Testo, Lenzkirch, Germany). After 1 week of incubation within the desiccator, filters were transferred to DG18 agar plates, and incubation was prolonged at an a_w_ of 0.96 for up to 1 month. Survival of fungal developmental structures, growth, and bursting of hyphal tips were evaluated and documented using a stereomicroscope (Zoom AZ-100; Nikon, Amsterdam, The Netherlands) within 30 min after transfer and after 1 and 2 days of incubation. The fungi were considered to have survived if hyphae reinitiated growth or the conidia germinated. These conidia could be originating from stage I and stage II or be newly formed at stage VI. A flowchart of this experimental setup is depicted in Fig. 2. Experiments were performed using at least two technical replicates and three biological replicates.

**TABLE 1:** Developmental stages exposed to changes in a_w_

Developmental stage no. (description)	Species	Culture time (h)^*a*^
I (dormant conidia)	A. niger, C. halotolerans, and P. rubens	0
II (swollen conidia)	A. niger, C. halotolerans, and P. rubens	7–8
III (germlings)	A. niger and P. rubens	18
	C. halotolerans	24
IV (microcolonies without aerial hyphae)	A. niger and P. rubens	24
	C. halotolerans	32–36
V (microcolonies with aerial hyphae)	A. niger and P. rubens	32–36
	C. halotolerans	44–48
VI (sporulating microcolonies)	A. niger and P. rubens	48–50
	C. halotolerans	48–60

a Time needed to reach the indicated developmental stage on DG18 agar.

### Fluorescence microscopy.

A Zeiss Axioplan II microscope, a Zeiss Plan NeoFluar 40×/0.75 objective, and a blue BP450-490 (FT510, LP520) excitation filter were used for fluorescence microscopy. Images were captured with a Zeiss AxioCam MRc digital camera run by Zeiss Axiovision 4. A 0.1 mM solution of the fluorescent dye quinolinium, 1-1′-[1,3-propanediylbis[(dimethyliminio)-3,1-propanediyl]]bis[4-[(3-methyl-2(3H)-benzothiazolylidene)methyl]]-, tetraiodide (TOTO-1; Molecular Probes, Breda, The Netherlands) in dimethyl sulfoxide (DMSO) was used as a stain of dead colonies. Colonies without aerial hyphae, grown on top of PC filters on DG18 agar (see above), were placed in a desiccator containing a saturated NaCl solution (75% RH) for 45 or 105 min. Filters were placed back on DG18 agar and stained for 25 min by using 2 μM TOTO-1 in ACES buffer (45, 46). A 1-cm^2^ square of the filter and the underlying DG18 agar was placed on top of an objective glass and covered with a coverslip. Colonies grown on a PC filter and killed with 70% alcohol or a 20-min steam treatment (47) served as a control.

### Cryo-SEM.

Small rectangular 5- by 5-mm blocks of agar medium topped with a filter were transferred to copper cups for snap-freezing in nitrogen slush. They were glued to the copper surface with frozen tissue medium (KP-Cryoblock; Klinipath, Duiven, The Netherlands) and sputter coated 3 times for 1 min each by using a gold target. Cryo-scanning electron microscopy (cryo-SEM) was done with a JEOL 5600LV scanning electron microscope (JEOL, Tokyo, Japan) equipped with an Oxford CT1500 Cryostation. Electron micrographs were taken at an acceleration voltage of 5 kV.

## RESULTS

### Minimal and optimal water activities for fungal growth.

**FIG 3:**
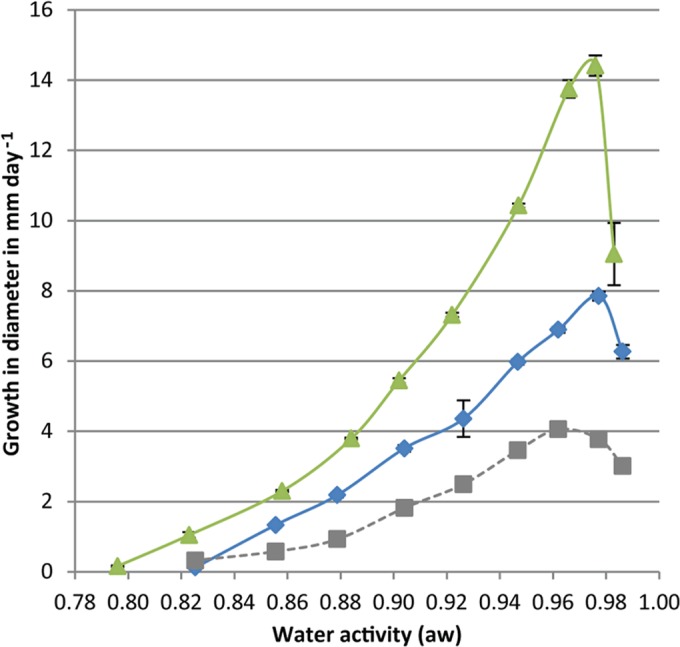
Growth (change in colony diameter) of A. niger (green triangles), C. halotolerans (gray squares) (data derived from the work of Segers et al. [40]), and P. rubens (blue diamonds) on MEA with 0 to 50% glycerol.

The minimal and optimal a_w_ values for growth of A. niger, C. halotolerans, and P. rubens were determined. Colony diameters of A. niger, C. halotolerans, and P. rubens colonies were monitored 3 times a week during a 3-week period on MEA plates with a_w_ values of 0.75 to 0.99 (Fig. 3). A. niger was the fastest-growing species, with an optimal growth speed of 14.4 mm day^−1^, while the optimal growth speed of P. rubens was 7.9 mm day^−1^. C. halotolerans has a growth speed of 4.1 mm day^−1^ as derived from the work of Segers et al. (40). These optimal growth speeds were observed at a_w_ values of 0.96 to 0.98. The minimal a_w_ values needed to support growth of A. niger, C. halotolerans, and P. rubens were 0.80, 0.82 (40), and 0.82, respectively.

### Survival during periods of dynamic a_w_.

A. niger, C. halotolerans, and P. rubens were tested for the ability to survive during periods of defined lowered a_w_, which were obtained using closed environments containing a saturated salt solution. Filters overlying DG18 agar medium were inoculated with freshly harvested conidia (stage I) and cultured at an a_w_ of 0.96 until conidia were swollen (stage II), conidia formed germ tubes (stage III), or microcolonies formed without aerial hyphae (stage IV), with aerial hyphae (stage V), or with conidium-forming conidiophores (stage VI) (Table 1; Fig. 1). The filters with these developmental stages were removed from agar and transferred to 33%, 58%, 75%, and 84% RH. No growth was observed during incubation in the desiccator at RHs of 33 to 75%, while growth continued in the desiccator at RHs above 84% for all developmental stages of the 3 species.

**FIG 4:**
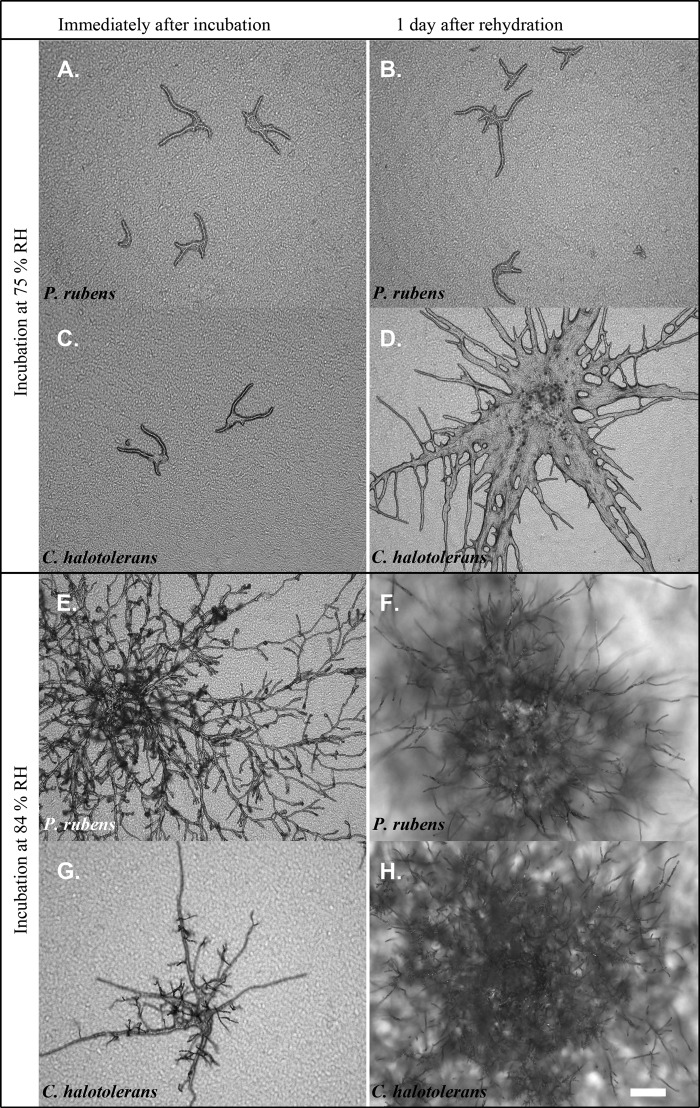
Branching hyphae of P. rubens (stage IV) (A, B, E, and F) and C. halotolerans (C, D, G, and H) immediately after incubation for 1 week at 75% RH (A and C) or 84% RH (E and G) and after subsequent incubation on DG18 agar for 1 day (B, D, F, and H). Bar = 100 μm.

After 1 week of incubation, survival was assessed by removing the filters from the desiccators and placing them on DG18 agar with an a_w_ of 0.96. The response of the fungi was monitored for up to 1 month. Incubation for 2 weeks to up to 1 month resulted in no more survival as determined 2 days after removal from the desiccator. Developmental stages II to V did not reinitiate growth at an a_w_ of 0.96 after a 1-week incubation at 33% RH. In contrast, about 50% of the conidia (stage I) of the 3 species germinated after transfer to medium with an a_w_ of 0.96. Similarly, germination of newly formed conidia was observed in the case of conidium-producing colonies (stage VI). Similar results were obtained after incubation at 58% RH. However, in this case, more conidia from stages I and VI germinated than those after incubation at 33% RH. Notably, stage V colonies of C. halotolerans also showed some growth at an a_w_ of 0.96 after exposure to 58% RH. All developmental stages of C. halotolerans incubated for 1 week at 75% RH showed survival after transfer of the filters to medium with an a_w_ of 0.96. Incubation of A. niger and P. rubens at 75% RH gave results similar to those obtained after incubation at 33% and 58% RH, except that more conidia of stages I and VI germinated. Thus, A. niger and P. rubens hyphae were unable to reinitiate growth after incubation at 75% RH (Table 2). Figure 4 shows examples of C. halotolerans and P. rubens after 1 week at 75% or 84% RH. P. rubens (stage IV) did not survive for 1 week at 75% RH (Fig. 4A and B), but in contrast, C. halotolerans did (Fig. 4C and D). Both fungi continued to grow during the period of 1 week at 84% RH, and both formed conidiophores (Fig. 4E and G). They showed strongly increased growth after 1 day of being rehydrated on DG18 agar (Fig. 4F and H).

**TABLE 2:** Survival of developmental stages of A. niger, C. halotolerans, and P. rubens after 1 week of incubation at 33, 58, 75, or 84% RH and subsequent 1 to 2 days of incubation on DG18 agar

RH (%) during 1 week of incubation	Developmental stage^*a*^	Survival^*b*^		
		A. niger	C. halotolerans	P. rubens
33	I	++	++	++
	II	−	−	−
	III	−	−	−
	IV	−	−	−
	V	−	−	−
	VI	+++	+++	+++
58	I	++++	++++	++++
	II	−	−	−
	III	−	−	−
	IV	−	−	−
	V	−	+	−
	VI	+++	+++	+++
75	I	++++	++++	++++
	II	−	++++	−
	III	−	++++	−
	IV	−	++++	−
	V	+	++++	−
	VI	++++	++++	++++
84	I	++++	++++	++++
	II	++++	++++	+++
	III	+++	++++	+++
	IV	+++	++++	+++
	V	++++	++++	++++
	VI	++++	++++	++++

a The developmental stages comprised dormant conidia (I), swollen conidia (II), germlings (III), and microcolonies without aerial hyphae (IV), with aerial hyphae (V), and with conidium-forming conidiophores (VI).

b Survival was qualified as no survival (−), <5% survival (+), <50% survival (++), >50% survival (+++), and >95% survival (++++).

**FIG 5:**
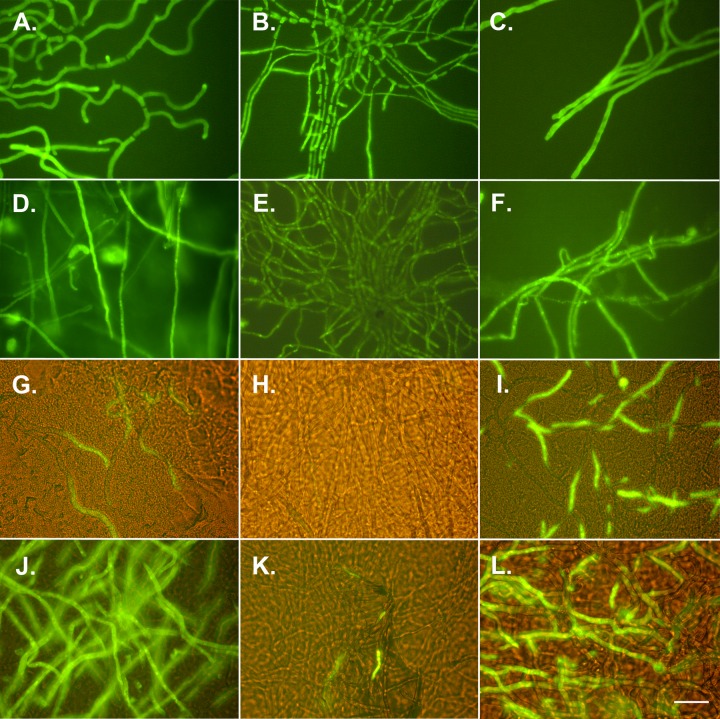
TOTO-1 staining, an indication of dead fungal structures, of A. niger (A, D, G, and J), C. halotolerans (B, E, H, and K), and P. rubens (C, F, I, and L) after treatment with steam for 20 min (A to C) or after exposure to 70% alcohol (D to F), to 75% RH for 45 min (G to I), or to 75% RH for 105 min (J to L). Bar = 20 μm.

Staining with TOTO-1 showed that some and most hyphae of A. niger and P. rubens were killed after 45 min (Fig. 5G and I) and 105 min (Fig. 5J and L), respectively, of exposure to 75% RH. In contrast, C. halotolerans showed no or little fluorescence after these exposures (Fig. 5H and K), indicating that most hyphae survived the treatment.

**FIG 6:**
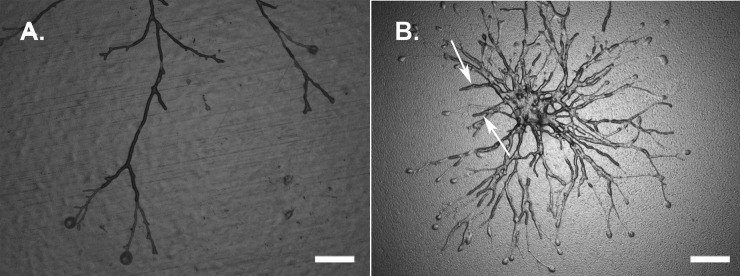
(A and B) Leading hyphae of P. rubens growing at 84% RH had burst after transfer to DG18 agar (a_w_ = 0.96). (B) New hyphae formed that originated from the center of the colony (white arrows) when the colony was left to grow for another 24 h after being transferred to DG18 agar. Bars = 100 μm (A) and 200 μm (B).

As mentioned above, growth of the colonies of the 3 species continued, albeit at a lower rate, during incubation at 84% RH and subsequent transfer to DG18 agar. However, 14.7% and 7.5% of all hyphal tips of microcolonies, including small branching hyphae, burst directly after the transfer for A. niger and P. rubens, respectively (Fig. 6). In contrast, hyphal tips of C. halotolerans hardly (0.2%) showed rupture. Most bursting hyphae were found at the periphery of the colony (Fig. 6A), while hyphae at the center of the microcolony resumed growth, as indicated with arrows (Fig. 6B).

## DISCUSSION

Relative humidity (RH) can change considerably during and between days in indoor situations. As a result, indoor fungi not only have to be able to survive periods of low RH but also have to be able to resume growth within the time frame of favorable RH. In this study, we compared the capacities of A. niger, C. halotolerans, and P. rubens to grow during static or dynamic water activity (a_w_) regimens.

A. niger grew at an a_w_ of 0.80 at 25°C, which was lower than the minimal a_w_ of 0.82 for C. halotolerans and P. rubens. The latter data confirm earlier findings (23, 40, 48, 49), although A. niger has been reported to grow even at an a_w_ of 0.78. The latter value was obtained at the optimal growth temperature of 30°C (23).

A controlled decrease in a_w_ was used to address the responses of A. niger, C. halotolerans, and P. rubens to dynamic a_w_ conditions. To this end, conidia were inoculated onto filters overlying DG18 medium (a_w_ = 0.96) and cultured until conidia had swollen or germinated or until microcolonies had formed without aerial hyphae, with aerial hyphae, or with conidium-forming conidiophores. The filters were then transferred to 84%, 75%, 58%, and 33% RH (a_w_ = 0.84, 0.75, 0.58, and 0.33). Conidia germinated at 84% RH but not at lower RHs, as could be expected according to the data presented above. Yet some or even all spores that had been exposed to the lower-RH conditions germinated when they were retransferred to DG18 agar after 1 week. The percentage of conidia that germinated increased with increasing RHs used during the 1-week incubation. This is interesting because conidia of Aspergillus fumigatus that had been dried slowly at room temperature survived for a year, with full germination (50). In our study, some of the conidia of the different fungal species lost their viability after a relatively quick shift from 96% to 33% RH and a week-long incubation, which indicates that the speed of change in RH is an important factor in survival even for survival structures, such as conidia. Alternatively, the extent of drying may be a factor that improves survival of spores. Ascospores of Talaromyces macrosporus and Neosartorya fischeri survive better when stringently dried (down to 0% RH) than when dried in ambient air (which is typically 40 to 60% RH as measured with a hygrometer) (51). This can be explained by the large amounts of compatible solutes that result in a glassy state at water contents below 2 to 3%. The mobility of molecules is very low in this state, thus suppressing the incidence of detrimental chemical reactions (e.g., with reactive oxygen species) (52). Environmental conditions during conidium formation are also important for survival of asexual spores. In preliminary experiments, we used MEA instead of DG18 agar, and this indicated less survival after a 1-week incubation period at 75% RH for C. halotolerans. It may well be that colonies grown on DG18 agar had adapted to the lower a_w_ of this medium and transferred this adaptation to the spores (53). It may also be that the glycerol in DG18 medium is taken up and serves as a compatible solute that helps the fungus and its conidia to survive during periods of low RH (24, 51).

**FIG 7:**
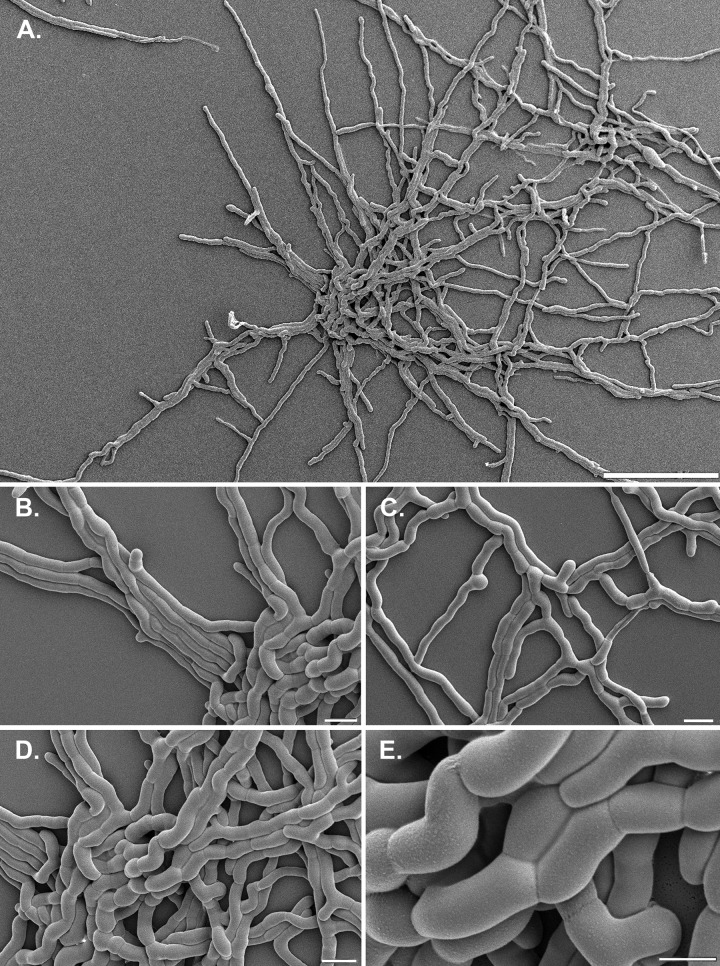
Cryo-SEM images of C. halotolerans, showing a 1-day-old colony growing on DG18 agar on top of a 0.1-μm-pore-size polycarbonate filter. The microcolony (A) can be seen with strengthened cells in the center (B, D, and E), while bundles of hyphae stretch out as leading hyphae (B and C). Bars = 100 μm (A), 10 μm (B to D), and 5 μm (E).

This study showed that the capability to grow at low a_w_ does not always reflect survival during changes in RH. All developmental stages of C. halotolerans were able to survive for 1 week at 75% RH. This was not the case for A. niger, despite its ability to grow at a lower steady-state a_w_. Notably, increasing water availability also affected survival. C. halotolerans hyphae had a much lower bursting incidence after transfer from 85% RH to medium with an a_w_ of 0.96 than those of A. niger and P. rubens. This was probably due to the suddenly increased a_w_ causing water influx into the hyphae. This may be due to differences in accumulation of compatible solutes between the species. Alternatively, the structure of the cell wall at the hyphal tips may differ. A more rigid cell wall at tips of C. halotolerans hyphae would more easily overcome a transfer from low to high water availability. At the same time, such a cell wall would restrict growth speed (54), which agrees with the lower growth speed of this species than those of A. niger and P. rubens. The rigidity of the C. halotolerans cell wall may be due to the presence of melanin. Nonlinear spectral imaging (NLSI) microscopy indicates that the cell wall of vegetative hyphae of C. halotolerans contains melanin, while melanin is absent in P. rubens (our unpublished results). Formation of enlarged cells with strengthened pigmented cell walls in the center of the colony (55), as well as formation of bundles of hyphae, may also help cells to overcome sudden changes in humidity (Fig. 7).

The ability of C. halotolerans to cope with dynamic water availability is probably related to the ecological niche of this fungus. Cladosporium species grow on leaves and are therefore called phylloplane fungi (56, 57). The available water for fungi growing on leaves is highly dynamic and is influenced by changing temperature, dew formation, sunlight, and rain. It is interesting that the indoor environment is also characterized by changes in humidity during the day. It has been shown that phylloplane fungi can restore growth after minutes to hours of rehydration after drying for 2 to 3 weeks (56). Furthermore, these fungi have to withstand large amounts of UV radiation from direct sunlight. Damage from UV radiation is prevented by melanin within the cell walls of conidia and hyphae (58–60). The protection against UV radiation is of less concern in indoor environments, which are generally darker aside from the small amount of UV passing through open windows. However, melanin can help in protection against reactive oxygen species or other reactive molecules present in building materials.

This study shows for the first time, to our knowledge, that steady-state a_w_ measurements, for so long the hallmark to determine the xerotolerance of a fungus, are not enough to predict the response of a fungus to humidity dynamics. The indoor environment is characterized by periods of lowered a_w_ as well as sudden increases in water availability, and fungal cells have to deal with both types of change. It has to be expected that the adaptability of a fungal species to deal with humidity dynamics is important for the predominance of the species on the relevant building material. Fungi may even be able to influence the (micro)environment by using enzymes or other excretions. For example, germinating conidia of P. rubens are known to produce an extracellular matrix on gypsum (61).
